# Comment on Coelho-Junior et al. Protein Intake and Frailty in Older Adults: A Systematic Review and Meta-Analysis of Observational Studies. *Nutrients* 2022, *14*, 2767

**DOI:** 10.3390/nu14224879

**Published:** 2022-11-18

**Authors:** William B. Grant

**Affiliations:** Sunlight, Nutrition and Health Research Center, P.O. Box 641603, San Francisco, CA 94164-1603, USA; wbgrant@infionline.net

The systematic review by Coelho-Junior et al. found that frail older adults consumed significantly less animal-derived protein than healthy people [1]. One reason that was suggested for this finding was that animal-based proteins have a 90% digestibility rate compared with a 50% rate for plant-based proteins. However, checking the reference for this statement [2], it was found that the authors noted that combining various plant-based proteins to provide a more favorable amino acid profile could increase the digestibility rate. Another reason was suggested: that animal proteins have higher branched-chain amino acid content.

However, the authors omitted the most important reason: animal products are important sources of vitamin D and vitamin D reduces risk of frailty. In 2011, a cross-sectional analysis of 25-hydroxyvitamin D [25(OH)D] concentration among 2107 white men and women in the UK reported that the amount of animal products in the diet significantly affected serum 25(OH)D concentrations [3]. Daily mean vitamin D intakes were 3.1 µg (95% confidence interval, CI, 3.0–3.2 µg) for meat eaters, 2.2 µg (95% CI, 2.1–2.4 µg) for fish eaters, 1.2 µg (95% CI, 1.1–1.3 µg) for vegetarians, and 0.7µg (95% CI, 0.6–0.8 µg) for vegans. The geometric mean 25(OH)D concentrations were 76.4 nmol/L (95% CI, 74.7–78.2 nmol/L) for meat eaters, 74.3 nmol/L (95% CI, 70.1–78.8 nmol/L) for fish eaters, 66.9 nmol/l (95% CI, 64.1–69.8 nmol/L) for vegetarians, and 55.9 nmol/L (95% CI, 51.0–61.3 nmol/L) for vegans. Similarly, a study of 22 Finnish vegans and 15 non-vegetarians found vegans had a mean 25(OH)D_2_ concentration of 27 nmol/L (25th and 75th percentiles: 19 and 36 nmol/L, respectively) and a 25(OH)D_3_ concentration of 31 nmol/L (25th and 75th percentiles: 15 and 41 nmol/L, respectively); meanwhile, non-vegetarians had a mean 25(OH)D_2_ concentration of 2 nmol/L (25th and 75th percentiles: 2 and 3 nmol/L, respectively) and a 25(OH)D_3_ concentration of 90 nmol/L (25th and 75th percentiles: 75 and 105 nmol/L, respectively) [4].

Animal protein is primarily muscles. Vitamin D is stored in muscles as 25(OH)D. A study using primary rat muscle fibers found that 25(OH)D is absorbed in mature muscle cells and held there by vitamin D-binding protein [5]. Furthermore, 25(OH)D stored in muscles helps maintain serum 25(OH)D concentrations when vitamin D production declines or ceases in winter [6,7].

Vitamin D deficiency is an important risk factor for frailty. A review discussed the genomic and nongenomic mechanisms whereby vitamin D increases muscle strength and reduces risk of frailty [8]. In in 2013, a cross-sectional study of frailty among 1504 community-dwelling elderly European men reported an adjusted relative odds ratio per 1 standard deviation 25(OH)D decrease of 1.89 (95% CI, 1.30–2.76) [9]. It also found an adjusted relative odds ratio per 1 standard deviation parathyroid hormone (PTH) increase of 1.24 (95% CI, 1.01–1.52). PTH concentrations are inversely correlated with 25(OH)D concentrations, with the PTH-to-25(OH)D ratio increasing with increasing age [10]. A meta-analysis found a pooled-risk estimate of frailty syndrome per 25 nmol/L increment in serum 25(OH)D concentration of 0.88 (95% CI, 0.82–0.95) in the six cross-sectional studies and 0.89 (95% CI, 0.85–0.94) in the four prospective cohort studies [11].

It is recognized that vegans have a risk of vitamin D deficiency. They should consider supplementing with vitamin D_3_ to raise serum 25(OH)D concentrations to above 30 or 40 ng/mL [12,13]. Other health benefits include reduced risk of incidence and death from Alzheimer’s disease, many types of cancer, cardiovascular disease, COVID-19, type 2 diabetes mellitus, and hypertension [13].

Vegans do not consume animal products, so are unlikely to take vitamin D_3_ supplements, which are mostly made from UVB-irradiated sheep’s wool lanolin, so prefer vitamin D_2_ supplements. However, vitamin D_3_ supplements made from vegetable sources are now available and can be found through searching the internet. Unfortunately, vitamin D_2_, made from fungi or yeast, is not as beneficial as vitamin D_3_. For example, a review found that vitamin D_2_ supplementation did not reduce mortality rate [8 studies, HR = 1.04 (95% CI, 0.97–1.11)], in contrast to vitamin D_3_ supplementation, which did reduce mortality rate [14 studies, HR = 0.89 (95% CI, 0.9 = 80–0.99)] [14]. A trial involving 33 healthy adults given 50,000 IU/week vitamin D_2_ or vitamin D_3_ found vitamin D_3_ is approximately 87% more potent in raising and maintaining serum 25(OH)D concentrations and produces 2–3-fold greater storage of vitamin D than equimolar vitamin D_2_ [15]. A systematic review and meta-analysis found vitamin D_3_ intervention was more efficacious than vitamin D_2_ in improving vitamin D status (mean difference of 41 nmol/L [95% CI, 32–50 nmol/L]), and regulating PTH levels, irrespective of the participant demographics, dosage, and vehicle of supplementation [16].
